# Navigating Tuberculosis in Pregnancy and Lactation: A Review of Maternal and Neonatal Considerations

**DOI:** 10.3390/diseases14030102

**Published:** 2026-03-11

**Authors:** Tiago Lima, Sandra Trigo, Eduarda Silveira, Gabriela Jorge da Silva, Sara Domingues

**Affiliations:** 1 Faculty of Pharmacy, University Coimbra, 3000-458 Coimbra, Portugal; 2 CNC-UC—Center for Neuroscience and Cell Biology, University Coimbra, 3004-517 Coimbra, Portugal; 3 CIBB—Centre for Innovative Biomedicine and Biotechnology, University Coimbra, 3004-548 Coimbra, Portugal; 4 Vasco da Gama Research Centre (CIVG), Department of Veterinary Sciences, Vasco da Gama University School, 3020-210 Coimbra, Portugal; 5 CERNAS—Research Center for Natural Resources, Environment and Society, 3045-601 Coimbra, Portugal

**Keywords:** tuberculosis, *Mycobacterium tuberculosis*, congenital tuberculosis, pregnancy, breastfeeding

## Abstract

Tuberculosis (TB) is an infectious disease caused by *Mycobacterium tuberculosis*. Despite the availability of effective treatments and advances in diagnostic methods, TB remains the leading cause of death from infectious disease globally, with its incidence tending to increase. Pregnant women constitute a population group with particular characteristics, as the diagnosis and treatment of certain conditions can be challenging. Early diagnosis and monitoring of TB by a multidisciplinary team are crucial to guide treatment and reduce complications. Congenital TB, although uncommon, is a serious complication that should be assessed in neonates, especially when the mother has previously been diagnosed with the disease. First-line anti-TB drugs are considered safe during pregnancy and lactation. In contrast, second-line drugs have a less well-established safety profile during breastfeeding, and the available evidence regarding their excretion in breast milk remains limited; therefore, their use requires individualised risk-benefit assessment. Data on this specific population group are limited, as physiological changes during pregnancy alter the pharmacokinetics/pharmacodynamics (PK/PD) of drugs and the inclusion of pregnant women in clinical trials remains contentious. Routine TB screening in prenatal care, particularly in high-prevalence regions, is crucial to improving maternal and neonatal outcomes. This narrative review was based on a structured search of PubMed, Scopus, and Web of Science (January 2000–June 2025), using the keywords tuberculosis, *Mycobacterium tuberculosis*, pregnancy, and breastfeeding. Eligible articles included original studies, reviews, and international guidelines.

## 1. Introduction

Tuberculosis (TB) may present as tuberculosis infection (TBI), characterised by the presence of viable bacilli without clinical symptoms and without transmissibility, although individuals remain at risk of progression to active disease. In immunocompetent adults, the lifetime risk is estimated at 5–10% [1,2]. In contrast, in neonates and young children, particularly infants under one year, historical cohort data indicate a substantially higher risk of progression to active disease in the first years after infection, with estimates often reported well above adult rates, reflecting age-dependent vulnerability [3,4]. Active TB represents the symptomatic and transmissible form of the disease and may be classified according to the site of infection, with pulmonary TB being the most common and infectious form, while extrapulmonary disease affects organs such as lymph nodes, pleura, bones, the central nervous system, or the genitourinary tract, especially in immunocompromised individuals [5,6,7]

To combat the worldwide TB epidemic, the WHO has proposed a strategic plan that aims to reduce incidence by 80% and fatalities by 90% by 2030. However, as the COVID-19 epidemic has affected access to TB diagnosis and treatment, making it more challenging to achieve these objectives, the task can be daunting [6,8].

During pregnancy, anatomical and physiological changes characteristic of this period may influence susceptibility to tuberculosis infection and progression to active disease. Although earlier reports suggested no significant differences, more recent evidence indicates that pregnant individuals may face an increased risk of developing active TB, particularly in the postpartum period [9,10]. However, during pregnancy, negative clinical outcomes can occur in both the pregnant woman and the foetus, depending on the location and severity of the disease, the response to anti-TB therapy, the gestational age at diagnosis, and co-infection with HIV. Therefore, early diagnosis and treatment are essential [11,12].

Immune function changes during pregnancy, potentially leading to a state of immunosuppression, which can influence the clinical manifestations of active TB and the sensitivity of diagnostic methods. The WHO has therefore recommended screening for four symptoms, namely cough, fever, nocturnal hyperhidrosis and weight loss. However, this screening is neither specific nor sensitive and, whenever possible, other diagnostic tools, such as chest X-rays and molecular diagnostics, should be employed to improve detection and diagnosis of the disease [11].

TB treatment during pregnancy should be monitored by a multidisciplinary team, including obstetricians and neonatologists, microbiologists, and other healthcare professionals. The goal is to define the appropriate treatment, minimise complications and negative clinical outcomes, prevent vertical transmission of TB, and avoid the development of resistance to anti-TB drugs, thereby ensuring positive outcomes for both the pregnant woman and the foetus [11,12].

Although congenital TB is an uncommon but serious condition, it is associated with a high mortality rate if not diagnosed and treated promptly. Early differential diagnosis of neonatal and/or pulmonary infections is therefore paramount. A high maternal bacillary burden, particularly in cases of disseminated or military TB, pulmonary TB with positive sputum smear microscopy, HIV co-infection, diabetes mellitus, severe malnutrition, smoking, and alcoholism in the pregnant woman are the main risk factors for foetal transmission of TB [13].

This review aims to analyse the current evidence on TB in pregnancy, congenital TB, and neonatal management, addressing diagnostic, therapeutic, and preventive strategies, as well as the impact of the disease on the pregnant woman, the newborn, and breastfeeding.

## 2. Methods

This study is a narrative review designed to synthesise current evidence on TB in pregnancy and congenital TB. A narrative approach was selected because the available literature consists mainly of case reports, small case series, heterogeneous observational studies, and guideline documents, which do not allow systematic comparison or quantitative synthesis.

A structured search was conducted at PubMed, Scopus, and Web of Science databases for articles published between January 2000 and June 2025. The search strategy combined the following keywords: “tuberculosis”, “*Mycobacterium tuberculosis*”, “pregnancy”, “maternal”, “neonatal”, and “breastfeeding”. For example, the PubMed search strategy included combinations such as: (“tuberculosis”[Title/Abstract] OR “*Mycobacterium tuberculosis*”[Title/Abstract]) AND (“pregnancy”[Title/Abstract] OR “maternal”[Title/Abstract] OR “congenital tuberculosis”[Title/Abstract] OR “neonatal”[Title/Abstract] OR “breastfeeding”[Title/Abstract]). In Scopus, search terms were combined using TITLE-ABS fields, for example: TITLE-ABS (tuberculosis OR “*Mycobacterium tuberculosis*”) AND TITLE-ABS (pregnancy OR maternal OR neonatal OR breastfeeding OR “congenital tuberculosis”). In Web of Science, a similar strategy was applied using Topic fields (TS): TS = (tuberculosis OR “*Mycobacterium tuberculosis*”) AND TS = (pregnancy OR maternal OR neonatal OR breastfeeding OR “congenital tuberculosis”). Additional sources were identified by manually screening reference lists and official guidelines. Additionally, conference abstracts without full published data and non-peer-reviewed preprints were not included. Pharmacokinetic-only studies were considered only when directly relevant to drug safety or dosing in pregnancy.

Eligible publications included original research articles, systematic reviews, narrative reviews, case reports, and international guidelines addressing TB in pregnant or lactating women, and its impact on maternal and neonatal outcomes. Publications were limited to English, Portuguese, and Spanish. Editorials and articles not directly addressing tuberculosis in pregnancy or lactation were excluded.

Two reviewers independently screened titles, abstracts, and full texts for eligibility. When discrepancies arose, they were resolved through discussion. Because congenital TB is extremely rare and reported evidence is heterogeneous, cases were synthesised qualitatively rather than aggregated numerically. To appraise the strength of evidence, priority was given to WHO guidelines, systematic reviews, and large cohort studies. When multiple sources addressed the same topic, preference was given to the most recent and highest-quality evidence. Narrative evidence was integrated where higher-level evidence was unavailable.

## 3. *Mycobacterium tuberculosis* Infection

### 3.1. Epidemiology

Although this review is not restricted to a specific geographic setting, epidemiological considerations are particularly relevant for both low-incidence European countries and high-burden regions, where diagnostic and management challenges may differ. Women of reproductive age in high-burden regions represent a particularly vulnerable population, especially in settings where TB overlaps with high HIV prevalence, poverty, and limited access to maternal health services [6].

Each year, an estimated 200,000 women develop active TB during pregnancy or the postpartum period, approximately 151,000 cases occurring during pregnancy and 49,000 in the postpartum period. Most of the cases are reported in sub-Saharan Africa and South-East Asia [14]. The risk of developing TB is 1.4 times higher during pregnancy and 1.9 times higher in the postpartum period compared to non-pregnant women [14,15].

Furthermore, TB is estimated to be responsible for 6–15% of all maternal deaths worldwide. Pulmonary TB is the most common form of the disease in this context; however, disseminated TB occurs in 5–10% of pregnant women with active disease and represents a particularly high risk of adverse outcomes [16]. Importantly, maternal disseminated or military has been described in a substantial proportion of reported congenital TB cases, which remain rare but carry high mortality. In large case series and systematic reviews, neonatal mortality rates of approximately 40–50% have been observed, with improved outcomes when early anti-tuberculosis therapy is administered. Although precise estimates of transmission risk are limited by the rarity of congenital TB, these data underscore the clinical significance of disseminated maternal disease for neonatal prognosis [17,18,19]. Despite its importance, the true global burden of TB in pregnancy remains underestimated, largely due to underdiagnosis, misclassification of maternal deaths, and absence of systematic reporting in antenatal and perinatal care programmes that contribute to significant data gaps, particularly in high-burden, resource-limited settings [9,15].

### 3.2. Transmission, Pathogenesis, and Immune Response

TB is transmitted when individuals with active pulmonary or laryngeal TB release aerosols containing viable bacilli, which are inhaled by susceptible contacts [12]. Understanding these transmission dynamics and host–pathogen interactions is particularly relevant in pregnancy, where physiological immune modulation may alter susceptibility, clinical presentation, and the risk of dissemination. The risk of transmission depends on host susceptibility, bacterial load, the persistence of the pathogen in the environment, and various contact-related factors such as duration, location, and frequency. Additionally, economic, social, and political conditions play an important role in TB transmission, as individuals in developing countries are not only more frequently exposed but also face limited access to healthcare [20].

Following inhalation, *M. tuberculosis* bacilli reaches the pulmonary alveoli, where it is initially phagocytosed by alveolar macrophages and resident dendritic cells through recognition of specific microbial components by phagocytic cell receptors, including Toll-like receptors (TLR-2, TLR-4, and TLR-9), C-type lectin receptors (such as the mannose receptor and Dectin-1), and cytoplasmic nucleotide-binding oligomerisation domain-like receptors [20,21].

**Figure 1:**
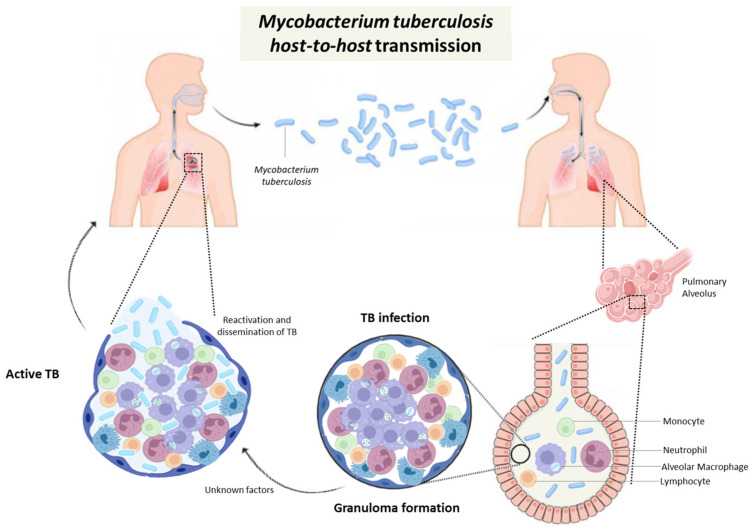
Schematic representation of the transmission, pathogenesis, and immune response to *Mycobacterium tuberculosis* infection. Transmission occurs via aerosolized droplets containing bacilli expelled by individuals with active pulmonary TB, which hosts inhale. Once in the alveoli, the bacilli are phagocytosed by alveolar macrophages, where they can survive and begin intracellular replication. The innate immune response triggers the recruitment of additional immune cells, including neutrophils, dendritic cells, and monocytes, leading to the formation of granulomas—organised immune structures aimed at containing the infection. Persistent bacilli within granulomas may result in TB infection; however, failure of immune control can lead to reactivation, tissue destruction, and the release of bacilli into the airways, thereby facilitating transmission to new hosts. Author-created schematic.

The pathogen, *M. tuberculosis* employs several mechanisms to survive within host cells and establish chronic infection, including interference with the maturation and acidification of phagosomes, and inhibition of apoptosis and necrosis. This allows intracellular replication, release of pro-inflammatory cytokines and the recruitment of immune cells that form early granulomas, as shown in Figure 1 [12].

If the initial defence mechanisms fail, antigen-presentation in regional lymph nodes activates adaptative immunity [21,22]. Although this restricts bacterial growth, infection is rarely eliminated, and granulomas become sites of long-term bacterial persistence. Moreover, there is a delay of approximately eight days between initial infection and the migration of T lymphocytes to the site of infection, which allows increased bacterial replication and limits the host’s ability to control the pathogen [20]. Loss of granuloma integrity may lead to caseous necrosis, tissue destruction, and dissemination to extrapulmonary sites, which is associated with higher risk in pregnant women due to immunological adaptations of gestation [23,24].

In pregnancy, a shift towards a tolerogenic immune state reduces Th1 responses, which may facilitate progression from TBI to active TB [25]. In the postpartum period, rebound immune activation further increases the risk of disease progression and dissemination [25,26,27]. These changes have important implications for maternal health, vertical transmission, and the development of congenital TB [16,25].

## 4. Congenital Tuberculosis

Gestational and congenital TB are relatively uncommon diseases. Consequently, there is limited epidemiological data in developed countries, with most information derived from case reports in the scientific literature. Nonetheless, it is estimated that, in developed regions, the incidence of gestational TB ranges from 4 to 39 new cases per 100,000 population annually. In contrast, in endemic areas, this number exceeds 60 new cases per 100,000 population [19].

Reports of congenital TB are even more limited, with fewer than 500 cases documented worldwide. This condition has a high mortality rate, estimated between 34% and 53%, and is more prevalent in low- and middle-income countries [17,28]. Pregnant women may present disseminated systemic or genital TB, including endometrial, cervical, or placental forms. Maternal genital TB has been associated with an increased risk of congenital transmission [18].

**Figure 2:**
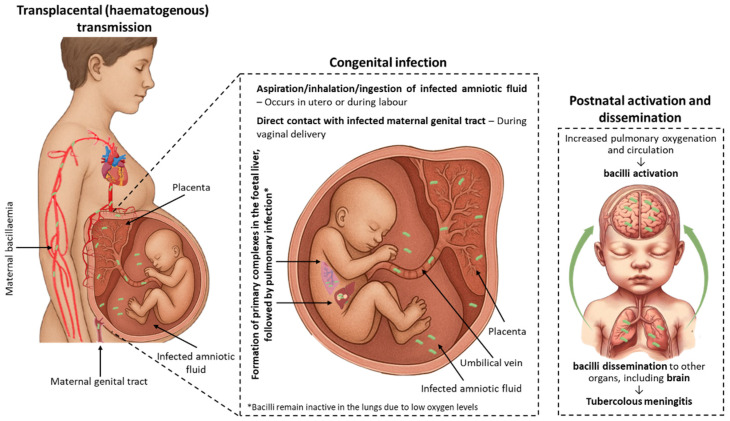
Vertical transmission and congenital progression of *Mycobacterium tuberculosis* infection. Maternal bacillaemia enables hematogenous spread of *M. tuberculosis*, with transplacental transmission occurring via the umbilical vein. The bacilli reach the foetal liver, forming primary complexes with periportal lymph node involvement. Hematogenous dissemination may also directly seed the foetal lungs. Additional intrauterine transmission may occur through aspiration, ingestion, or inhalation of infected amniotic fluid, leading to primary infection in the lungs and/or gastrointestinal tract. Postnatally, increased oxygenation and pulmonary circulation may activate bacilli persisting in a state of TBI in the lungs, enabling dissemination to other organs, including the brain, where tuberculous meningitis may develop. Author-created schematic.

Congenital TB is a rare, serious, and frequently under-reported condition. Its clinical manifestations are typically non-specific and may mimic other neonatal or congenital infections. Congenital TB, if left untreated, is associated with a high mortality rate, highlighting the importance of early and accurate differential diagnosis of neonatal or pulmonary infections, especially in high-burden countries [13]. As illustrated in Figure 2, congenital infection is believed to be acquired via maternal bacillaemia, with transmission occurring through the placenta, amniotic fluid, and/or maternal genital tract [13,29]. Vertical transmission, specifically haematogenous infection, occurs via the transplacental route. In such cases, *M. tuberculosis* crosses the placenta via the umbilical vein, leading to the development of primary complexes in the foetal liver with involvement of periportal lymph nodes, followed by pulmonary infection [13,30]. Foetal infection can be acquired in utero or during labour through aspiration, inhalation, or ingestion of infected amniotic fluid, resulting in primary complexes formation in the lungs or gastrointestinal tract. Less commonly, transmission may occur through direct contact with an infected maternal genital tract during delivery [13,30,31].

In the lungs, bacilli typically remain inactive during the foetal period and shortly after birth, becoming active only with the postnatal increase in oxygenation and pulmonary circulation. Once activated, the bacilli may disseminate to other organs, including the brain, potentially leading to tuberculous meningitis, a clinical situation generally associated with a worse prognosis [13,30].

### Clinical Manifestations

Clinical manifestations of congenital TB can emerge between 1 and 84 days after birth and are often non-specific or atypical. There are no pathognomonic clinical signs unique to the disease. Nevertheless, the most common symptoms include poor feeding, fever, irritability, failure to thrive, cough, dyspnoea, hepatosplenomegaly, lymphadenopathy, and abdominal distension. However, these symptoms may also occur in other infectious diseases such as bacterial pneumonia, sepsis, meningitis, or acute infantile hepatitis. As such, congenital TB should be considered and excluded through differential diagnosis [13,18].

Additionally, more severe clinical manifestations may occur, including meningitis, septicaemia, miliary pneumonia, disseminated intravascular coagulation, lethargy, jaundice (obstructive or not, due to hepatic involvement, occasionally progressing to liver failure), ascites, otitis media with or without mastoiditis, parotitis, osteomyelitis, paravertebral abscess, cold abscess, and papular or pustular skin lesions. Rarer manifestations include apnoea, facial paralysis, vomiting, cyanosis, seizures, petechiae, and haemophagocytic syndrome [13].

## 5. Diagnosis

The diagnosis of TB relies on a combination of clinical, microbiological, immunological, and imaging approaches, but several of the traditional methods have important limitations [32,33]. The microbiological culture remains the gold standard, since it not only allows identification of the *Mycobacterium* species but also enables antitubercular drug susceptibility testing, however its long turnaround time restricts its clinical utility. Smear microscopy is inexpensive and widely accessible but has low sensitivity and cannot distinguish *M. tuberculosis* from non-tuberculous mycobacteria. Similarly, the tuberculin skin test (TST) shows poor accuracy in differentiating TBI from active disease and is influenced by prior Bacille Calmette-Guérin (BCG) vaccination [24,34,35]. In contrast, interferon-gamma release assays (IGRAs) offer higher specificity by using antigens absent from BCG and most non-tuberculous mycobacteria, and they have become increasingly relevant for detecting TBI [24,34,36].

Radiological imaging, particularly chest X-ray, remains the standard initial assessment in suspected pulmonary or extrapulmonary TB. While computed tomography (CT) offers greater sensitivity, it is not routinely recommended due to higher radiation exposure and cost. Other imaging modalities, such as nuclear imaging techniques like positron emission tomography/computed tomography (PET/CT), can detect active disease (except in the kidneys and central nervous system), assess disease extent, and monitor treatment response, but are associated with higher radiation and costs, and are often unavailable in many tuberculosis-endemic countries [24,34,37]. Imaging innovations such as lung ultrasonography (LUS), in contrast, is non-invasive, radiation-free, and cost-effective. Emerging studies highlight its potential for diagnosing TB, especially in high-risk populations such as individuals living with HIV, children, pregnant women, and patients in resource-limited settings, where conventional imaging may be limited [38].

In recent years, molecular methods that detect *M. tuberculosis* nucleic acids (DNA/RNA) by nucleic acid amplification tests (NAATs) have become central to TB diagnosis. Molecular WHO-recommended rapid diagnostic tests (mWRDs), such as Xpert MTB/RIF Ultra and Truenat, are recommended as the initial diagnostic tests for individuals with signs and symptoms of TB, in preference to smear microscopy, because they provide rapid results and detect rifampicin resistance [39,40]. Their sensitivity and specificity are high in smear-positive disease, while sensitivity is lower in smear-negative specimens [39]. In smear-positive samples, NAATs should be used to confirm *M. tuberculosis* complex and assess rifampicin resistance; a discordant pattern (smear-positive/NAAT-negative) should prompt consideration of nontuberculous mycobacterial infection or other causes of false-positive smear [39,40]. Examples include Xpert MTB/RIF Ultra for rifampicin resistance detection and Deeplex Myc-TB for species identification, resistance profiling and lineage determination [41,42].

### 5.1. Diagnosis of Tuberculosis During Pregnancy

The nonspecific symptomatology and the physiological immunosuppression characteristic of pregnancy complicate the timely diagnosis of TB, which is crucial for initiating treatment. These factors are mainly due to the inhibition of T-helper 1 pro-inflammatory responses, leading to the absence of symptoms, the establishment of asymptomatic infection, or increased susceptibility to new infections and TB reactivation [12,43].

In high-burden settings, systematic symptom-based screening during antenatal care is often implemented. However, in low-incidence countries, TB screening during pregnancy is generally risk-based, focusing on women with epidemiological risk factors such as recent exposure, HIV infection, immunosuppression, or origin from endemic regions [44,45].

In the presence of clinical signs suggestive of active TB, the diagnostic evaluation should prioritise microbiological and radiological assessment, including chest X-ray and bacteriological testing (smear microscopy and/or NAAT). Immunological tests such as TST or IGRA may be useful to identify latent infection in selected cases but cannot confirm active disease and should not delay microbiological investigation [12,24,34,39,43].

TST and IGRA are not diagnostic tests for active TB disease, but immunological tests used to detect TBI. Their results must always be interpreted in conjunction with the clinical presentation and investigations for active disease. While pregnancy does not significantly alter their interpretation, both tests have important limitations, and neither can distinguish TBI from active TB. IGRA may offer greater specificity than TST, particularly in BCG-vaccinated individuals, but neither test can confirm or exclude active TB on its own. For this reason, any positive TST or IGRA requires further assessment, including symptom screening, clinical evaluation, and chest imaging [12,13,19].

Nonetheless, TST remains a safe and valid test, producing a positive result 2 to 12 weeks after exposure to the infectious agent. It is indicated in symptomatic pregnant women who had close contact with an individual with infectious TB, or are at high risk of developing active TB during pregnancy [12,19].

However, a positive result in either the TST or IGRA indicates exposure to the TB bacillus but does not distinguish TBI from active infection [12,19,24]. Thus, active TB must be ruled out before initiating treatment for TBI. This should involve clinical assessment of the pregnant woman and a chest X-ray. The level of ionising radiation exposure from this imaging method is below the threshold associated with adverse foetal effects and is performed using an abdominal lead shield [12]. Delaying the X-ray until after the first trimester may be considered depending on the epidemiological risk and clinical presentation. However, in pregnant women living with HIV or those with recent contact (within the previous two years) with an individual with active or documented TB, chest X-ray should be performed regardless of gestational age [12,46].

### 5.2. Diagnosis of Congenital Tuberculosis

Congenital TB is a challenging condition to diagnose, partly due to the difficulty in distinguishing it from TB acquired in the early postnatal period. To this end, diagnosis should be made by direct microscopic examination using Ziehl-Neelsen staining. To collect samples, non-invasive methods such as gastric aspirates, induced sputum, tracheal aspirates in mechanically ventilated neonates, cutaneous lesions, or ear secretions are preferred [13,18,19]. In addition to the non-invasive methods already referenced, stool specimens should also be considered. The WHO recommends processing stool samples for Xpert MTB/RIF or Ultra testing as a feasible and non-invasive alternative in children when respiratory specimens are difficult to obtain. Although stool testing is mostly described for paediatric pulmonary TB, its use in neonates, including in the context of congenital or early-life TB, may provide a practical, less invasive adjunctive diagnostic pathway. Given the high risk and rapid progression of TB in neonates, stool-based molecular testing represents a valuable option warranting inclusion in diagnostic algorithms when available [16,47,48]. In particular cases, more invasive methods may be required, including ascitic fluid, cerebrospinal fluid, or pleural fluid sampling [18,19].

Imaging plays a crucial role in the diagnostic evaluation of suspected congenital tuberculosis. Chest radiography should be performed in all suspected cases and may reveal miliary patterns, consolidations, or mediastinal lymphadenopathy. Abdominal ultrasound is also recommended to assess hepatosplenomegaly, ascites, or hepatic lesions, including evidence of a primary hepatic complex. In the presence of a miliary pattern on chest radiography or neurological manifestations, further evaluation of the central nervous system is warranted, including lumbar puncture and contrast-enhanced magnetic resonance imaging (MRI) to exclude tuberculous meningitis or intracranial tuberculomas [3,16,17,18,49].

In addition, a complete blood count should be performed, serum C-reactive protein (CRP) levels assessed, and a biochemical analysis of liver function carried out, to rule out anaemia, thrombocytopenia, leukocytosis, and elevations in transaminases and CRP. The TST should also be performed, although its sensitivity is low in neonates, and because IGRA is not an approved diagnostic method in neonates due to the immaturity of their immune system [13,19,31].

In cases where the newborn presents severe clinical manifestations, particularly those affecting the central nervous system, lumbar puncture and cranial ultrasonography should be considered. In cases of suspected meningitis, diagnostic workup should include cytochemical analysis, culture, and PCR in the cerebrospinal fluid. If the diagnosis remains inconclusive but clinical suspicion is high, more invasive diagnostic procedures such as bronchoscopy, transbronchial biopsy, liver biopsy, or lymph node biopsy may be necessary [13,18,19].

Furthermore, when congenital TB is suspected, histological examination of the placenta should be carried out to detect the presence of granulomas, alongside culture of the bacillus, smear microscopy, and acid-fast staining [30].

Current diagnostic criteria for distinguishing congenital TB from TB acquired during the neonatal period are based on the revised Cantwell criteria [49]. These include the presence of tuberculous lesions in the neonate along with at least one of the following: onset of symptoms during the first week of life; a presence of a primary hepatic complex or caseating hepatic granulomas; identification of *M. tuberculosis* infection in the placenta or maternal genital tract; or exclusion of postnatal transmission through thorough contact tracing and adherence to current infection control guidelines. These criteria remain the most widely cited framework for diagnosing congenital TB and provide a structured approach in clinical practice [30,31,49].

From a practical perspective, congenital TB should be suspected in neonates born to mothers with active, untreated, or recently diagnosed TB who present with early systemic symptoms such as respiratory distress, fever, hepatosplenomegaly, poor feeding, or signs of sepsis unresponsive to broad-spectrum antibiotics, particularly within the first weeks of life [16,30,31,49]. The minimum diagnostic evaluation should include chest radiography and microbiological testing of appropriate specimens (e.g., gastric aspirates, respiratory samples, or stool NAAT when available), with further assessment for extrapulmonary involvement guided by clinical findings [13,16,18,19,47]. Given the high mortality associated with delayed treatment, empiric anti-tubercular therapy should be considered when clinical suspicion is high, even if microbiological confirmation is pending [3,16,18,49].

## 6. Treatment

### 6.1. Treatment of Tuberculosis in Pregnant Women

The initiation of TB treatment during pregnancy should be assessed by a multidisciplinary team comprising obstetricians, microbiologists, pulmonologists, general practitioners, neonatologists, nursing staff, and public health authorities. Physiological changes in gestation alter the pharmacokinetics and pharmacodynamics (PK/PD), which may increase the risk of hepatotoxicity and inappropriate systemic drug exposures, putting both mother and foetus at risk, and may also contribute to the development of antimicrobial resistance. Careful monitoring allows optimisation of efficacy and safety while reducing adverse effects and preventing vertical transmission [11,50].

Standard first-line treatment for active TB consists of a combination of drugs, namely rifampicin (RIF) and isoniazid (INH), for a minimum duration of six months. During the initial two months, pyrazinamide (PZA) and ethambutol (EMB) are also administered. If PZA is excluded from the regimen, treatment should be extended to at least nine months. Once initiated, treatment should not be interrupted, and fixed-dose combinations facilitate adherence [11,30,46].

Baseline drug susceptibility testing is important; however, initial treatment should begin with the four first-line drugs until results are available [46].

Preventive therapy with INH (IPT) during pregnancy is recommended when there is a high risk of progression from TBI to active disease, such as recent contact with an infectious case or HIV infection with low CD4 counts [30,51]. The main aim of IPT in this context is to reduce the likelihood of haematogenous dissemination of TB to the placenta. INH should be administered at 5–10 mg/kg/day, without exceeding a maximum daily dose of 300 mg, for six months, regardless of the gestational trimester. Concurrently, pyridoxine supplementation at 25 mg/day should be initiated. In case of intolerance or INH resistance, RIF at 10 mg/kg/day (maximum 600 mg/day) for four months is an alternative [30,51,52,53]. However, treatment of TBI in pregnancy requires individualised assessment. In most cases, treatment can be safely deferred until two to three months postpartum to reduce the risk of hepatotoxicity, but in high-risk women therapy should not be delayed, even in the first trimester [30,51,52,53].

Overall, the use of first-line drugs (INH, RIF, EMB, and PZA) during pregnancy is considered safe for both the mother and the foetus [30]. All these drugs cross the placenta, but no significant teratogenic effects have been reported when used appropriately [54,55,56]. Table 1 summarises their main safety considerations, adverse effects and monitoring in pregnancy.

#### 6.1.1. First-Line Drugs

##### Isoniazid

INH is considered safe in pregnancy and remains a cornerstone of treatment. However, its administration is associated with some adverse effects, particularly on liver function, which requires careful monitoring. Treatment should be discontinued if there is a 3- to 5-fold increase in transaminases or bilirubin levels, especially in women over 35 years of age or in those receiving concomitant therapy with RIF, as this combination increases the risk of hepatic impairment. Therefore, before initiating treatment, the pregnant woman’s general clinical condition (e.g., fever, malaise, anorexia, nausea, vomiting, and other nonspecific symptoms), as well as liver function, should be assessed. Hepatic monitoring should be conducted every two weeks during the first eight weeks of treatment, and weekly during the first two weeks if there is a pre-existing chronic liver disease. Thereafter, monthly monitoring is recommended [30,46,55].

INH is also associated with neurological toxicity, which may manifest as peripheral sensory neuropathy, optic neuritis, seizures, or dizziness. This is due to competitive inhibition of the coenzymes pyridoxal phosphate and pyridoxamine, derived from vitamin B6, which are essential cofactors in the synthesis of amines that function as neurotransmitters. Thus, pregnant women taking isoniazid should be supplemented with pyridoxine at a minimum dose of 25 mg/day in pregnant women and 1 mg/kg/day in newborns to reduce the likelihood of peripheral neuropathy [11,46,55].

##### Rifampicin

RIF is widely used in pregnancy and is generally safe. RIF induces microsomal hepatic cytochrome P450 enzymes, thereby altering the metabolism of other drugs. Its administration may be associated with several adverse effects, including cutaneous reactions, fever, nausea, vomiting, hepatic injury and, in rare cases, bleeding episodes, in which case treatment must be discontinued. Moreover, when administered intermittently (i.e., less than three times a week), RIF may cause haemolytic anaemia, renal failure or dyspnoea, and such regimens should be closely supervised. In addition, administration in the final weeks of pregnancy may increase the risk of postpartum haemorrhage in both the mother and newborn, and vitamin K supplementation may therefore be indicated [46,55,56].

##### Pyrazinamide

Despite limited data regarding the teratogenicity of PZA, its use during pregnancy is recommended by the WHO, as it allows for a reduction to a 6-month treatment regimen. Additionally, liver function should be regularly monitored, since PZA administration is associated with the risk of the occurrence of hepatitis. Moreover, its active metabolite inhibits the renal excretion of uric acid, potentially causing arthralgia. Other reported side effects include flushing, photosensitivity, anorexia, nausea, and pruritus [11,46,55].

##### Ethambutol

Although EMB crosses the placenta freely, it is considered safe in pregnancy and some studies have shown that its administration during the first trimester of pregnancy is not associated with increased risks of spontaneous abortion, preterm birth, stillbirth or congenital malformations. However, prolonged use or high serum concentrations may lead to retrobulbar neuritis [46].

#### 6.1.2. Second-Line Drugs

Multidrug-resistant TB (MDR-TB), defined as resistance to both INH and RIF, presents a major therapeutic challenge during pregnancy [6,53,57]. Treatment of TB during pregnancy requires careful risk–benefit assessment when considering second-line drugs, as their known and potential adverse effects, including teratogenicity and foetal toxicity, are generally more severe than those of first-line agents. For example, streptomycin is contraindicated in pregnancy due to the risk of foetal auditory and vestibular damage, underscoring the importance of cautious drug selection in this population [11,30,46].

Given the significant challenges posed by MDR-TB during pregnancy, treatment should be individualised and closely monitored. It typically includes at least four second-line antitubercular agents, in addition to PZA. When the pregnant woman is clinically stable and presents minimal radiological evidence of disease, therapy may be deferred until the second trimester to avoid potential teratogenic risk during organogenesis associated with drug exposure during the first trimester [11,46].

Among the second-line options, several classes of anti-TB drugs have been considered, each with distinct implications for pregnancy. Thus, fluoroquinolones, especially levofloxacin and moxifloxacin, are considered to have a relatively better safety profile among second-line agents. However, their use during pregnancy or lactation is generally discouraged unless the benefits clearly outweigh the risks. Ciprofloxacin, while less effective against *M. tuberculosis*, is administered orally, is rapidly absorbed and achieves maternal serum concentrations that approximate those found in amniotic fluid and breast milk, facilitating estimation of foetal and neonatal drug exposure. Nonetheless, prolonged administration at high doses has been associated with congenital musculoskeletal abnormalities, requiring close monitoring [33,46,57,58].

Macrolides, such as clarithromycin, may exhibit synergistic effects with PZA and proton pump inhibitors, but due to a higher associated risk during pregnancy compared to other macrolides, their use is not recommended [46].

The combination of amoxicillin and clavulanic acid has demonstrated bactericidal activity against *M. tuberculosis* and is occasionally used prophylactically in cases of preterm rupture of membranes between 26 and 36 weeks of gestation. However, no data are available regarding its use in early pregnancy [46,59].

Para-aminosalicylic acid (PAS) has been associated with congenital malformations when administered during the first trimester and may induce gastrointestinal and cutaneous adverse effects, limiting its use to cases where no safer alternatives are available [46,60].

Ethionamide is contraindicated due to demonstrated teratogenicity in animal studies, including omphalocele, anencephaly and cleft palate, and should therefore be avoided during pregnancy [30,46,59].

Cycloserine and terizidone are associated with central nervous system side effects, including dizziness, insomnia, psychosis and seizures, especially at high doses, warranting extreme caution if considered during pregnancy [46,59].

Lastly, bedaquiline (BDQ) a relatively recent option for drug-resistant TB, has limited pregnancy data. Reported concerns include low birth weight, QTc prolongation and elevated hepatic enzymes, necessitating regular electrocardiogram and biochemical monitoring, as well as serum electrolyte evaluation throughout therapy [59,61,62].

In summary, treatment of MDR-TB during pregnancy must be individualised, prioritising maternal survival while minimising foetal risk. Whenever second-line therapy is necessary, decisions should be documented, maternal and foetal monitoring intensified, and neonatal follow-up arranged.

### 6.2. Treatment of Congenital Tuberculosis

The management of congenital tuberculosis follows the same principles as paediatric TB treatment, with weight-adjusted regimens and prolonged therapy to ensure eradication of bacilli and prevention of relapse. Current WHO guidelines recommend a six-month regimen, consisting of an intensive phase of two months with INH, RIF, PZA, and EMB, followed by a continuation phase of four months with INH and RIF [13,16,18,30,63]. In cases of disseminated disease or central nervous system involvement, therapy may be extended to 9–12 months [13,16,18]. Congenital TB is often severe; therefore the shorter 4-month regimen endorsed by WHO for non-severe paediatric TB generally does not apply in this setting [16]. Pyridoxine supplementation (5–10 mg/day) is recommended to prevent neurotoxicity related to INH [16,30], and adjunctive corticosteroids are indicated in congenital TB meningitis or severe miliary disease [13,30].

As drug resistance in the maternal strain directly impacts neonatal management, susceptibility testing is crucial, and resistant cases may require individualised regimens with second-line drugs, although evidence on their safety in neonates is limited [16,59,62]. Given the high mortality associated with untreated congenital TB, prompt initiation of appropriate anti-tubercular therapy is essential once the diagnosis is suspected or established [18,28].

## 7. Implications of Tuberculosis in Pregnancy and Breastfeeding

### 7.1. Implications in Pregnancy

Infection with *M. tuberculosis* during gestation is associated not only with a threefold increase in the risk of antenatal hospitalisation, anaemia, and caesarean delivery, but also with a ninefold increase in the likelihood of spontaneous abortion and a six-fold increase in perinatal death. Furthermore, TB doubles the risk of preterm birth and low birth weight. The risk of maternal mortality is particularly high in pregnant individuals with active TB who are living with HIV [12,56].

However, when active TB is diagnosed and treatment is initiated during the first trimester of pregnancy, the increased risk of preterm birth, low birth weight, maternal complications, and perinatal death is virtually eliminated compared with treatment initiation later in gestation [12].

### 7.2. Implications in Breastfeeding

The risk–benefit assessment of breastfeeding during TB treatment should ideally involve a multidisciplinary team including neonatologists, obstetricians, and pharmacologists particularly in complex cases or when second-line drugs are used [11,56]. In resource-limited settings, management may follow WHO-based guidance within primary care structures, focusing on maternal treatment adherence, infection control measures, and appropriate neonatal monitoring [12,44]. First-line anti-TB drugs are excreted into breast milk in very small amounts and are therefore not considered harmful to the infant, nor do they reach therapeutic levels to treat active or TBI in the neonate [11,12,56]. Serum concentrations of INH in breast milk result in an estimated infant exposure of approximately 0.1–0.2 mg/kg/day, 0.4–0.5 mg/kg/day for rifampicin, 0.02–0.07 mg/kg/day for pyrazinamide, and 0.08 mg/kg/day for ethambutol. These exposures are far below the recommended therapeutic doses for neonates, which range from 10 to 30 mg/kg/day depending on the drug. Consequently, breastfed infants are unlikely to receive sufficient drug to prevent or treat TB, and if prophylaxis or therapy is required, appropriate paediatric dosing must be administered with monitoring of plasma drug concentrations [12,56,64]. Although such low-level drug exposure is generally considered safe and unlikely to cause harm, recent studies have raised the theoretical concern that it could contribute to the selection of resistant *M. tuberculosis* strains in breastfed infants who become infected. However, this risk remains unconfirmed and further research is needed [65].

RIF administration may lead to orange-red discoloration of bodily fluids, including breast milk; however, this is harmless to both mother and infant and should be explained to the breastfeeding individual [46].

Breastfeeding may be initiated at least two weeks after starting anti-TB treatment, provided the mother is sputum-negative. In cases of continued infectivity, the use of a protective mask is advised [11,12]. To minimise drug exposure through milk, it is recommended that the mother takes her medication after breastfeeding, ideally when the infant begins its longest sleep period [12].

Although data on the excretion of fluoroquinolones in breast milk are limited, their use during lactation must be carefully evaluated due to the risk of cartilage damage [66,67]. In the case of macrolides, the estimated excretion of clarithromycin into breast milk is approximately 1.7% of the maternal weight-adjusted dose [68].

Regarding BDQ, this drug is also excreted in breast milk. Although human data remain limited, existing reports suggest that BDQ concentrations in human milk may exceed those found in maternal plasma, consistent with findings from preclinical animal studies [69]. Consequently, systemic exposure in breastfed infants may reach levels comparable to those in their mothers undergoing BDQ treatment. Given the limited evidence, breastfeeding is generally not recommended for individuals receiving BDQ, but decisions should be made on a case-by-case basis, weighing the potential risks and benefits [61,69].

Breastfeeding is contraindicated in cases of untreated active TB at the time of delivery, particularly in mothers living with HIV, those with active tuberculous lesions in the breast ducts or glands, or those with tuberculous mastitis. In such cases, milk from the affected breast must be discarded [12,56].

In practical terms, breastfeeding is generally compatible with first-line anti-TB therapy. When second-line drugs are required, particularly newer agents such as BDQ, an individualised risk–benefit assessment is necessary due to limited safety data. Breastfeeding is contraindicated in untreated active TB at delivery or in cases of active tuberculous mastitis. Practical implementation of these recommendations should be adapted to local epidemiology, drug availability, and healthcare resources [25,30,61,69].

## 8. Prevention of *Mycobacterium tuberculosis* Infection During Pregnancy and the Neonatal Period

The prevention of *M. tuberculosis* infection and its progression to active disease is essential to reduce maternal and neonatal morbidity and to achieve global TB control goals [70].

Beyond clinical management, prevention involves both non-pharmacological measures that limit exposure and transmission, and pharmacological approaches that reduce the risk of progression from infection to disease [71].

Non-pharmacological interventions remain fundamental, particularly in high-burden settings. Early identification of infectious cases, rapid initiation of treatment, and strict infection-control practices in maternity wards and neonatal units are essential. Adequate natural or mechanical ventilation, and the use of upper-room ultraviolet germicidal irradiation, where available, help reduce airborne transmission. Household-level measures, including improving ventilation, temporary distancing from infectious individuals, and mask use by the index case, are recommended until the infectious period declines. Screening of close contacts, particularly pregnant women and newborns, is crucial for timely detection of infection or early disease in high-burden settings or in the presence of specific risk factors (e.g., HIV infection, recent exposure, immunosuppression). In low-incidence settings, screening is generally implemented using a targeted, risk-based approach [71,72].

Pharmacological prevention, or tuberculosis preventive treatment (TPT), aims to reduce the risk of progression from infection to active TB. The WHO recommends TPT during pregnancy for women at high risk, such as those living with HIV or with recent exposure. Regimens considered safe include INH for 6 to 9 months or daily regimens combining RIF and INH, while weekly rifapentine and INH for 3 months is not recommended during pregnancy and should be reserved for the postpartum period [33,71]. Management of MDR-TB exposure remains individualised and follows specialist guidance [71].

Newborns exposed to maternal TB require early assessment and careful follow-up, and isoniazid prophylaxis may be initiated according to maternal infectiousness, treatment duration, and WHO recommendations. Subsequent evaluation with TST or clinical monitoring guides continuation or discontinuation of prophylaxis, as detailed in the treatment of congenital TB section [29].

Vaccination plays a key role in the prevention of infectious diseases, including TB. Currently, only the BCG vaccine, derived from attenuated *M. bovis* strains, is approved and is commonly administered at birth in countries with high TB prevalence. However, routine BCG vaccination is not universally implemented, and in low-incidence countries, it is often reserved for high-risk groups or specific circumstances [70,73]. In infants born to mothers with active TB, the timing of BCG vaccination remains debated across guidelines. The WHO recommends that vaccination be delayed until active TB disease has been excluded, as BCG is contraindicated in neonates with suspected or confirmed TB. Furthermore, in neonates under INH prophylaxis, there is no agreement on the optimal timing of BCG administration following treatment. WHO recommends administering BCG two weeks after completing INH prophylaxis, provided the TST is negative [29].

In conclusion, during pregnancy and the neonatal period, integrating infection-control practices, timely screening, targeted preventive treatment, and appropriate vaccination is essential to reduce the burden of maternal and congenital TB [46].

## 9. Discussion and Future Directions

Tuberculosis in pregnancy and congenital TB remain under-recognised by clinical entities despite their significant impact on maternal and neonatal outcomes. Evidence from recent reviews consistently shows substantial delays in diagnosis, challenges distinguishing congenital from early postnatal infection, and persistent gaps in maternal screening during pregnancy [13,29,30,31,32]. This review addresses these issues by providing an integrated overview of maternal, neonatal, and congenital TB, incorporating updated WHO recommendations, the evolving concept of the TB infection spectrum in pregnancy, and emerging neonatal diagnostic tools, including stool-based NAATs [16,24,40,45,47,74]. Additionally, it identifies areas where evidence remains scarce, particularly concerning congenital TB and newer therapeutic regimens during pregnancy.

However, the work also has inherent limitations. Congenital TB is extremely rare, and much of the available literature consists of heterogeneous case reports and small series, limiting the strength of epidemiological conclusions. Evidence on diagnostic approaches remains fragmented, with inconsistencies in reporting criteria and follow-up. As a narrative review, this manuscript may also be subject to selection bias despite the structured search strategy. These limitations reflect the broader challenges of researching a neglected condition and reinforce the need for robust, pregnancy-specific studies.

One promising avenue is host-directed therapy (HDT), which aims to modulate the host immune response in order to enhance bacillary clearance and limit tissue damage. Agents such as metformin, vitamin D and low-dose aspirin have been investigated as adjunctive therapies, with preliminary evidence suggesting benefits in immune priming. Nevertheless, robust clinical data are still lacking, and pregnancy-specific trials remain a priority [75,76,77,78].

Another important field is the repurposing of existing drugs. Several agents originally developed for other indications, including linezolid, clofazimine and fluoroquinolones, are now part of regimens for multidrug-resistant TB, and observational data suggest acceptable maternal and neonatal outcomes when newer agents such as BDQ or delamanid are included [62,79,80]. However, safety data remain limited, and certain regimens, particularly those containing pretomanid, are not currently recommended in pregnancy due to insufficient evidence [81]. Continued pharmacovigilance, pregnancy registries and targeted pharmacokinetic studies are therefore essential to refine risk–benefit assessments and to expand safe therapeutic options for pregnant women [62,79].

In addition, prospective pharmacokinetic and pharmacodynamic (PK/PD) studies stratified by gestational stage are urgently needed to guide appropriate dosing of first-line agents such as rifampicin, isoniazid, pyrazinamide and ethambutol. Physiological changes during pregnancy significantly affect drug absorption, distribution, metabolism and clearance, which may result in subtherapeutic exposure if standard dosing is used. Gestation-stratified dosing, combined with therapeutic drug monitoring, could ensure adequate maternal exposure while minimising foetal risk [50]. Similar work is especially needed for newer agents such as BDQ, for which current evidence in pregnancy remains limited to small cohorts, and where systematic evaluation of safety, placental transfer and long-term infant outcomes is still lacking [62,81].

Preventive strategies also represent a major research priority. While isoniazid monotherapy for 6–9 months has long been the standard, shorter regimens such as three months of weekly rifapentine plus isoniazid (3HP) or one month of daily rifapentine plus isoniazid (1HP) are increasingly adopted in the general population. However, data on their safety, tolerability and effectiveness in pregnant and postpartum women remain scarce. Dedicated studies are needed to establish whether these shorter regimens can safely improve adherence and reduce the risk of progression from TBI to active TB in this vulnerable group [71]. In parallel, long-acting injectable formulations of rifapentine–isoniazid are under development and may provide sustained protection throughout gestation and the postpartum period, offering a practical solution to improve adherence in maternal–infant pairs [50].

Finally, maternal immunisation strategies are likely to gain importance as new TB vaccines, such as M72/AS01E, advance into late-phase clinical trials. Although data on the safety of vaccination during pregnancy are not yet available, such approaches may ultimately provide dual protection for mothers and their infants in high-burden settings [70].

In addition to pharmacological innovations, addressing the social and structural barriers to TB care in pregnancy is essential. High-burden, resource-limited settings often face challenges such as limited access to diagnostic services, stigma, poverty, and migration-related vulnerabilities. Integrated, patient-centred care models that combine medical treatment with social support, community engagement, and proactive follow-up may improve adherence and outcomes in these populations [82].

Taken together, key priorities for future research include: (i) gestation-stratified pharmacokinetic and pharmacodynamic profiling of first-line and newer anti-TB drugs; (ii) safety and effectiveness of shorter preventive regimens in pregnant and postpartum populations; (iii) systematic evaluation of placental transfer and long-term infant outcomes associated with second-line therapies; (iv) standardisation of congenital TB diagnostic criteria and operational workflows; and (v) integration of context-adapted TB screening strategies within antenatal care systems. Addressing these priorities requires the responsible inclusion of pregnant and breastfeeding women in clinical research to ensure that future therapeutic advances are both safe and evidence-based.

## 10. Conclusions

TB during pregnancy remains a significant public health concern due to its association with adverse maternal and neonatal outcomes. Based on current evidence, several key clinical messages emerge:

(1) Early, risk-based screening and timely diagnosis are essential, particularly among high-risk populations such as women living with HIV, immunocompromised individuals, and those from TB-endemic regions.
(2) First-line anti-tubercular therapy is generally safe during pregnancy and breastfeeding when appropriately monitored, whereas second-line regimens require individualised risk–benefit assessment.
(3) Congenital TB, although rare, carries high morbidity and mortality and requires high clinical suspicion and prompt empiric treatment when suspected.
(4) Breastfeeding is usually compatible with first-line therapy, but management must be stratified when second-line drugs are used or when active untreated disease is present.
(5) The systematic exclusion of pregnant and breastfeeding women from clinical trials remains a critical gap, limiting evidence-based optimisation of dosing, shorter preventive regimens, and newer therapeutic agents.

Strengthening pregnancy-specific research, pharmacovigilance systems, and context-adapted screening strategies is essential to improve maternal and neonatal outcomes globally.

## Figures and Tables

**Table 1:** Pharmacological and safety considerations for first-line anti-TB drugs in pregnancy.

Anti-TB Drug	Dosage	Monitoring	Adverse Effects	Crosses Placenta	Comments
Isoniazid (INH)	5 mg/kg/day (max 300 mg)	Liver function (ALT/AST)	Hepatotoxicity, peripheral neuropathy, skin rash	Yes	Safe in pregnancy and breastfeeding; pyridoxine (25–50 mg/day) supplementation is recommended
Rifampicin (RIF)	10 mg/kg/day (max 600 mg)	Liver function (ALT/AST)	Hepatotoxicity, drug interactions, orange-coloured secretions	Yes	May reduce efficacy of hormonal contraceptives; vitamin K (10 mg/day) may be given in the last 2–4 weeks
Ethambutol (EMB)	15–25 mg/kg/day (max 1200 mg)	Visual acuity, liver function (ALT/AST)	Optic neuritis (rare at standard dose), hepatotoxicity	Yes	Considered safe in pregnancy; monitor vision regularly
Pyrazinamide (PZA)	20–30 mg/kg/day (max 1500 mg)	Liver function (ALT/AST), serum uric acid if symptomatic	Hepatotoxicity, arthralgia, nausea, skin rash	Yes	Recommended by the WHO; use in pregnancy varies by country (e.g., limited use in the U.S.)

## Data Availability

No new data were created or analyzed in this study. Data sharing is not applicable to this article.
